# Supplementary material for: Thymic iNKT single cell analyses unmask the common developmental program of mouse innate T cells
Source: Nat Commun. 2020 Dec 7;11:6238. doi: 10.1038/s41467-020-20073-8 (PMC7721697; doi:10.1038/s41467-020-20073-8)
Supplement: Supplementary file 1 — Supplementary Information [file 41467_2020_20073_MOESM1_ESM.pdf]

Supplementary Information

Thymic iNKT cells single cell analyses unmask the common developmental program of mouse innate T cells

S. Harsha Krovi[1], Jingjing Zhang[1], Mary Jessamine Michaels-Foster[1], Tonya Brunetti[1], Liyen Loh[1], James Scott-Browne[1, 2] and Laurent Gapin[1, 2]

[1] Department of Immunology and Microbiology, University of Colorado Anschutz Medical Campus, Aurora, CO, USA.
[2] Department of Biomedical Research, and Department of Pediatrics, National Jewish Health, Denver, CO, USA.

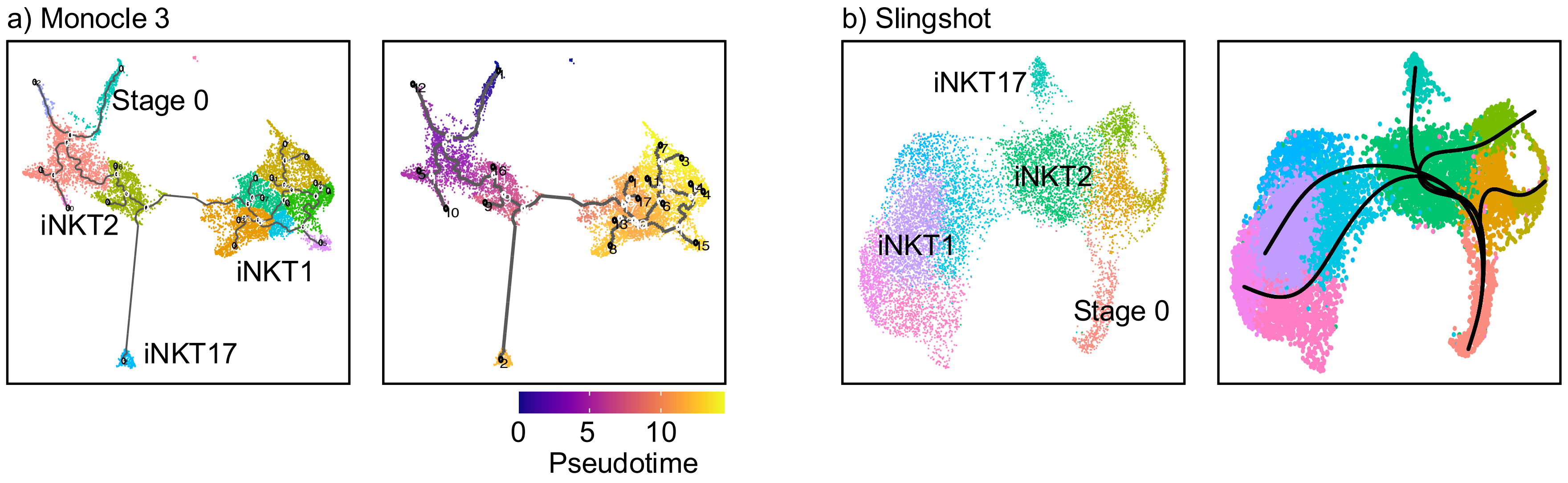

Supplementary Figure 1. Developmental trajectories of thymic iNKT cells. (**a**) Pseudotemporal trajectory was computed using the Monocle3 trajectory inference R package and displayed on a UMAP plot. Cells are colored by both cluster identity and along their computed pseudotime values. (**b**) Pseudotemporal trajectories computed using the Slingshot trajectory inference R package on a UMAP plot. Cells in cluster 0 were provided as the root node for both softwares since those cells represent the earliest iNKT cell precursors. The softwares then determine the differentiation paths that the stage 0 cells could take based on gene expression.

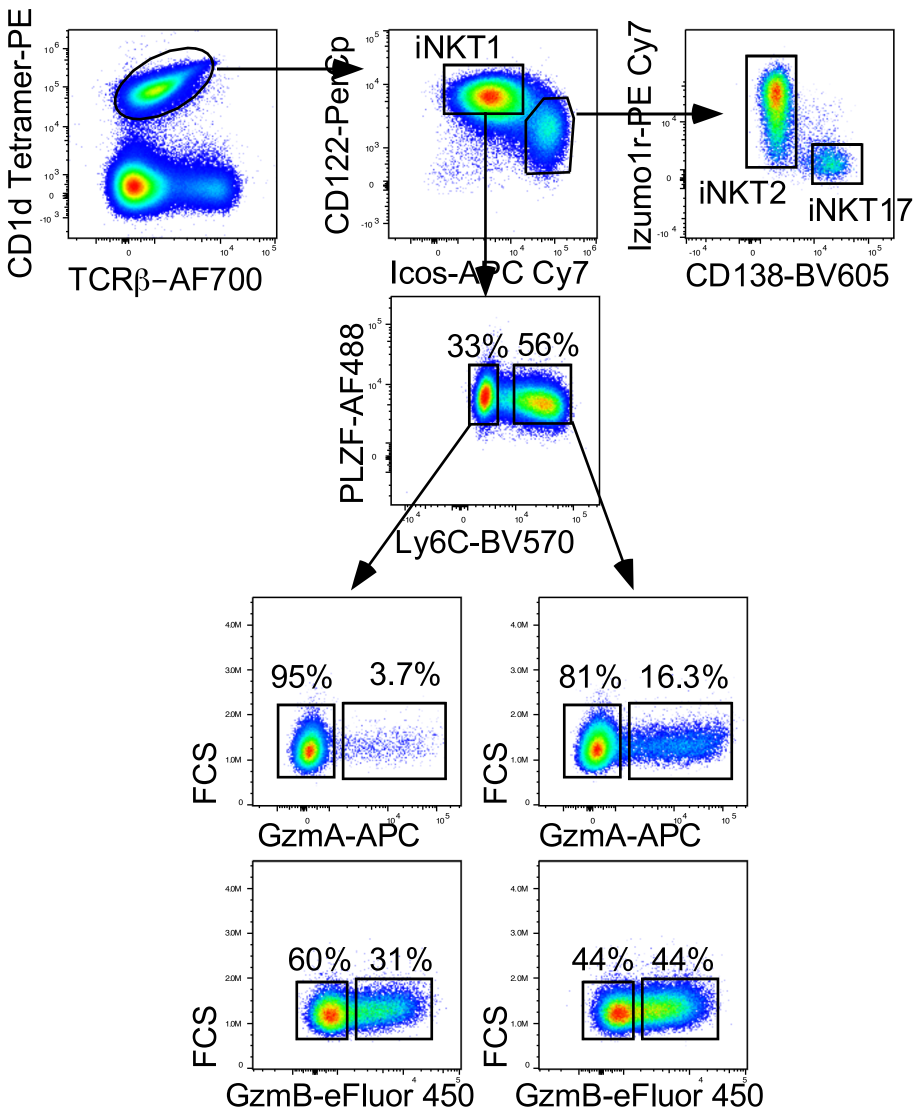

Supplementary Figure 2. Thymic Ly6C⁺ iNKT1 cells express higher amounts of GZMA and GZMB proteins than Ly6C⁻ iNKT1 cells. Representative flow cytometry plots depicting the gating strategy used to identify the various iNKT cell subsets in the thymus of 8-week-old C57BL/6 mice using the cell surface markers CD122, ICOS, Izumo1r and CD138. iNKT1 cells were subsequently separated based on Ly6C expression and expression of both GZMA and GZMB proteins in Ly6C⁻ and Ly6C⁺ iNKT1 are depicted. Data are representative of 2 independent experiments with similar results.

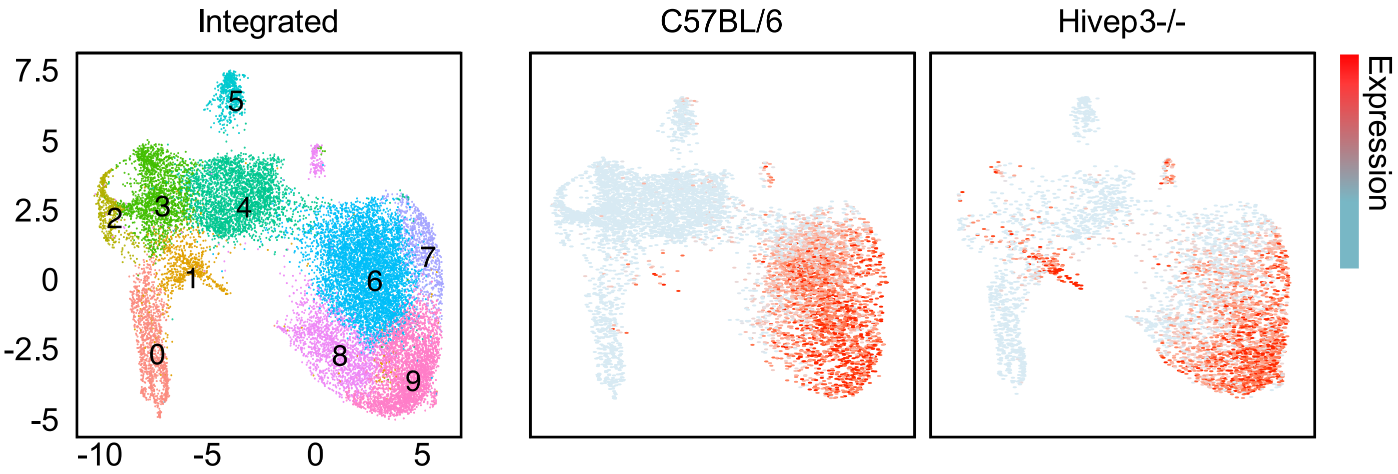

Supplementary Figure 3. ***Hivep3*$^{-/-}$ cluster 1 cells possess a mature iNKT1 gene signature.** Expression of selected iNKT1 gene signature (*Cd7*, *Dusp1*, *Ms4a4b*, *Il2rb*, *Fcer1g*, *Klhdc2*, *Klra1*, *Klf2*, *Klrb1a*, *Cd160* and *S100a4*) in the C57BL/6 and *Hivep3*$^{-/-}$ single cell RNA-seq samples. Each dot represents one cell and gene expression is plotted along a colorimetric gradient, with red corresponding to high expression.

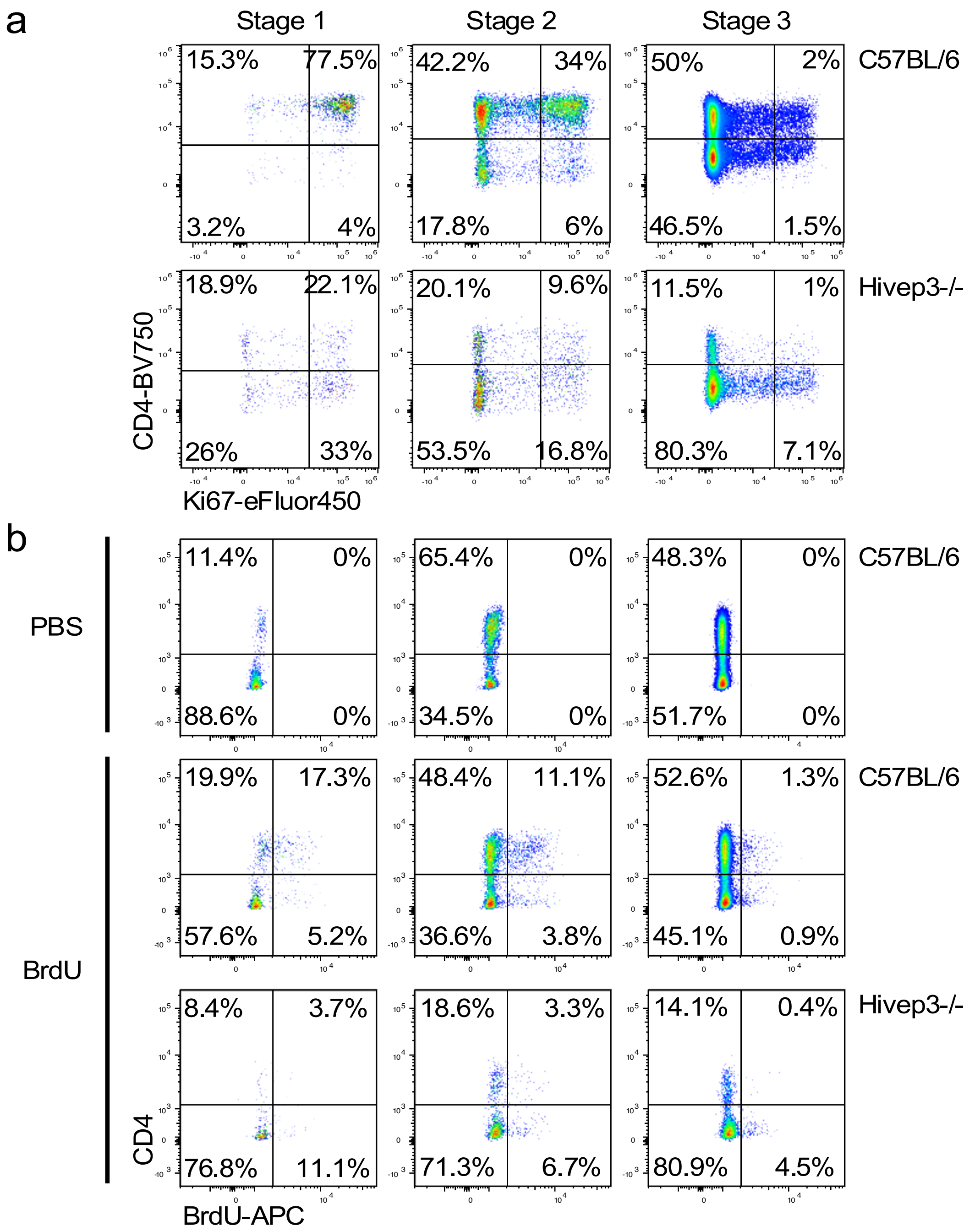

Supplementary Figure 4. *Hivep3*$^{-/-}$ iNKT cells display a reduced proliferative potential. (a) Representative flow cytometry plots depicting the expression of CD4 and Ki67 on the three developmental stages of iNKT cells (Stage 1, CD44^{-}NK1.1^{-}, stage 2, CD44^{+}NK1.1^{-} and stage 3, CD44^{+}NK1.1^{+}) in the thymi of 8-week-old C57BL/6 and *Hivep3*$^{-/-}$ mice. Data are representative of two independent experiments with 5-7 mice per group. 8-10 weeks old mice of both sexes were used. (**b**) Representative flow cytometry plots depicting the expression of CD4 and incorporation of BrdU by the three developmental stages of iNKT cells in the thymi of 8-week-old C57BL/6 and *Hivep3*$^{-/-}$ mice. Data are representative of one experiment with 3 mice per groups.

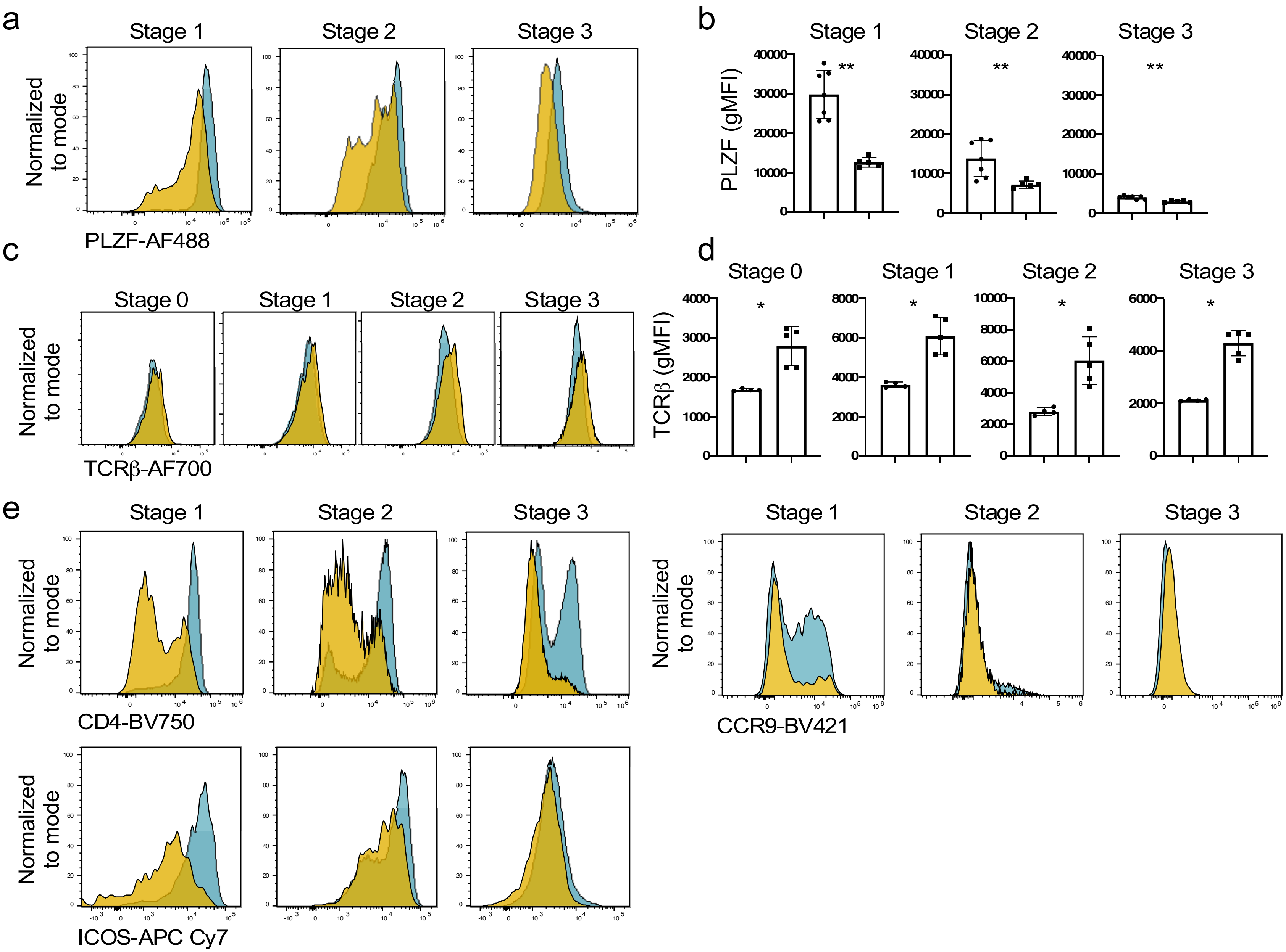

Supplementary Figure 5. Characterizing protein expression of *Hivep3*$^{-/-}$ iNKT cells. (**a**) Representative flow cytometry plots depicting the expression of PLZF on the three developmental stages of iNKT cells in the thymi of 8-week-old C57BL/6 and *Hivep3*$^{-/-}$ mice. (**b**) Quantification of PLZF expression (gMFI) in three different developmental stages. Stage 1 *p*=0.025, Stage 2 *p*=0.051, Stage 3 *p*=0.025 (**c**) Representative flow cytometry plots depicting the levels of expression of TCRβ by the four developmental stages of iNKT cells (Stage 0, CD24^{+}CD44^{-}NK1.1^{-}, Stage 1, CD44^{-}NK1.1^{-}, stage 2, CD44^{+}NK1.1^{-} and stage 3, CD44^{+}NK1.1^{+}) in the thymi of 8-week-old C57BL/6 and *Hivep3*$^{-/-}$ mice. (**d**) Quantification of TCRβ expression (gMFI) in four different developmental stages. Stage 0 *p*=0.0159 ,Stage 1 *p*=0.0159, Stage 2 *p*=0.0159, Stage 3 *p*=0.0159 (**e**) Representative flow cytometry plots depicting the expression of CD4, CCR9 and ICOS on the three developmental stages of iNKT cells in the thymi of 8-week-old C57BL/6 and *Hivep3*$^{-/-}$ mice.
Data are representative of two independent experiments with a total of 6 B6 and 5 Hivep3$^{-/-}$ mice per group. Data in the figure are mean ± SD with dots representing individual values. Source data are provided as a source data file.

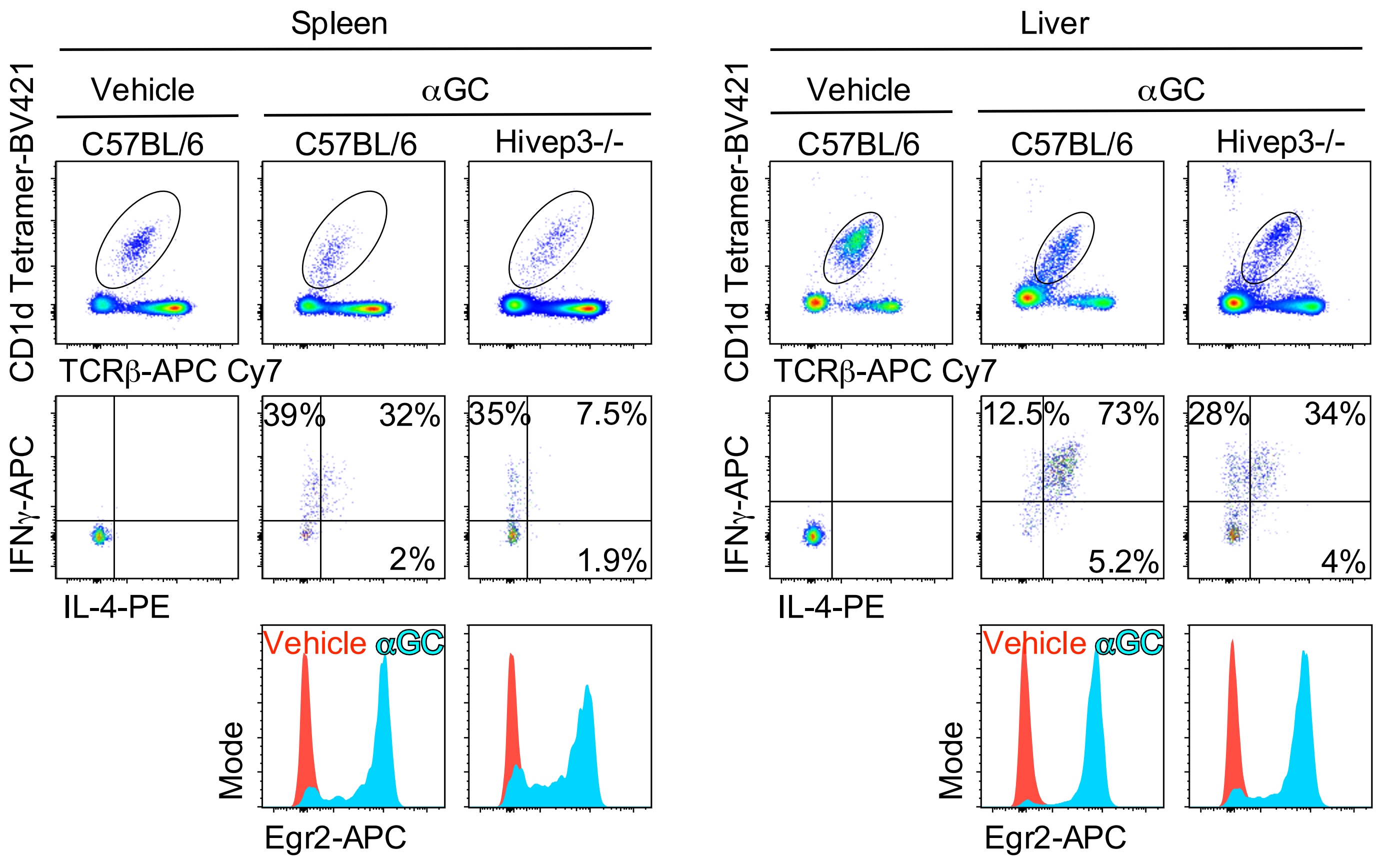

Supplementary Figure 6. Peripheral iNKT cells in *Hivep3*$^{-/-}$ mice are poorly responsive. iNKT cells from C57BL/6 and *Hivep3*$^{-/-}$ mice were isolated from the spleen and liver 90 minutes following i.p. administration of the agonist lipid αGC and stained intracellularly for IFNγ and IL-4. The cells were also stained for the transcription factor Egr2 (data representative of n = 3 mice per group).

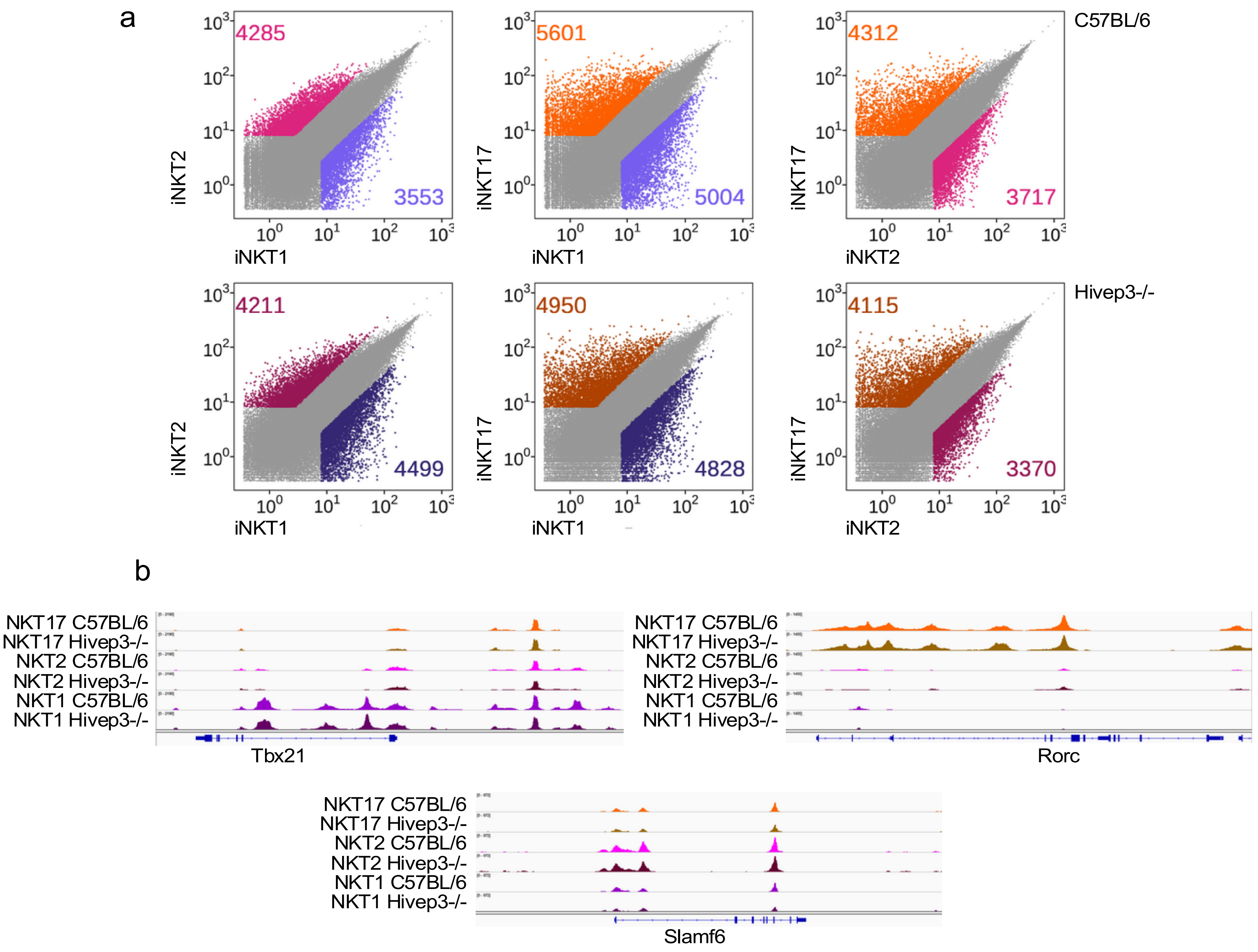

Supplementary Figure 7. Genome-wide chromatin landscapes readily identify iNKT subsets in both C57BL/6 and *Hivep3*$^{-/-}$ mice. (**a**) Scatterplots of mean ATAC-seq counts per peak comparing iNKT1, iNKT2 and iNKT17 subsets from C57BL/6 (top) or *Hivep3*$^{-/-}$ (bottom) mice. Pairwise contrasts were performed with limma and differentially accessible regions (colored) were filtered based on an FDR adjusted p-value of less than 0.1 and an estimated fold-change of at least 3. (**b**) Mean ATAC-seq coverage at the *Tbx21*, *Rorc* and *Slamf6* loci for ATAC-Seq tracks.

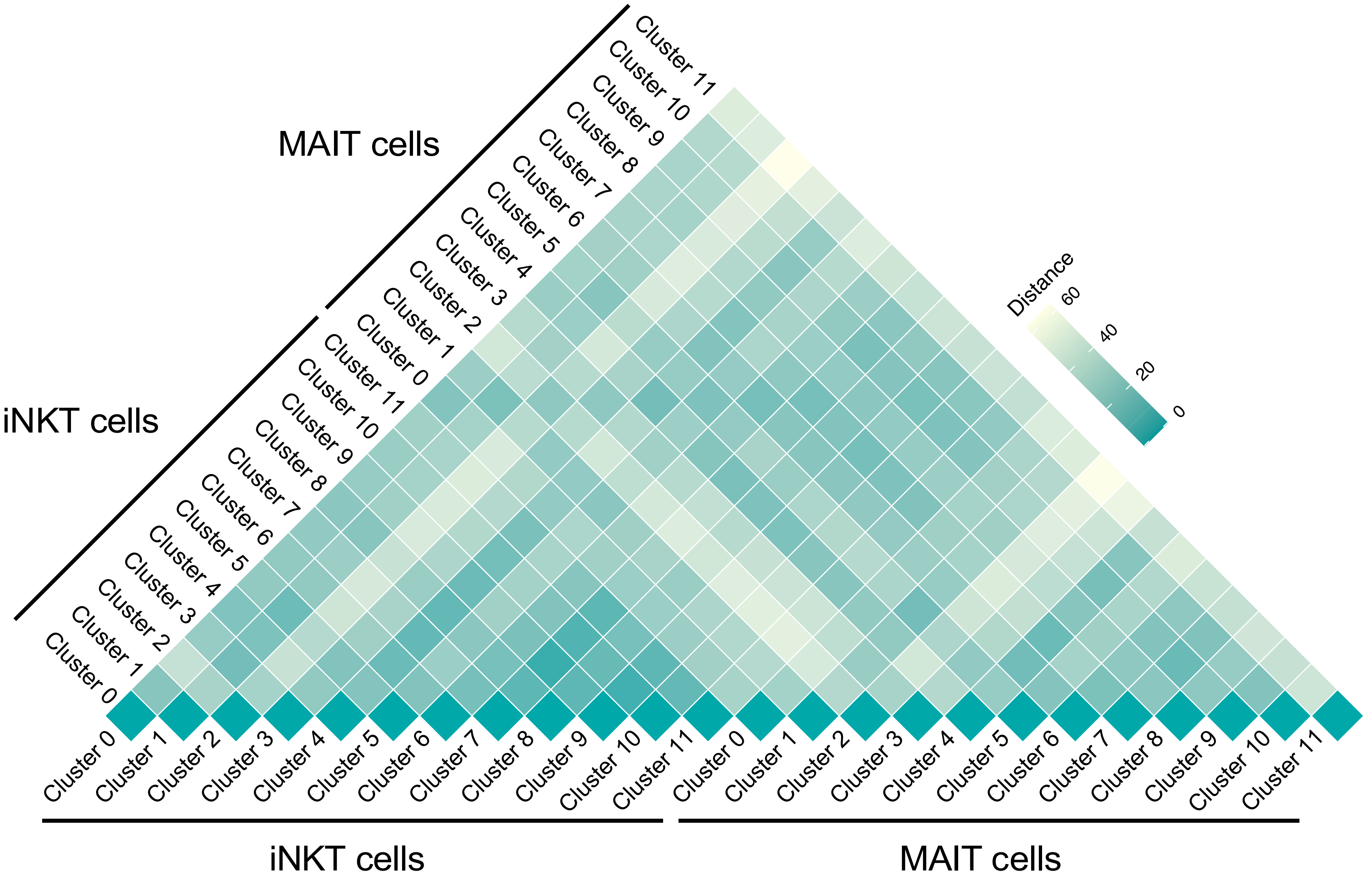

Supplementary Figure 8. iNKT and MAIT cells belonging to the same cluster possess greater transcriptional similarity to each other than to cells in other clusters. Average expression of all genes in cells belonging to a given cluster was used to calculate pairwise Euclidean distances across both iNKT and MAIT cells in all clusters. The distances were then plotted as a heatmap, with a darker shade corresponding to greater similarity between two groups.

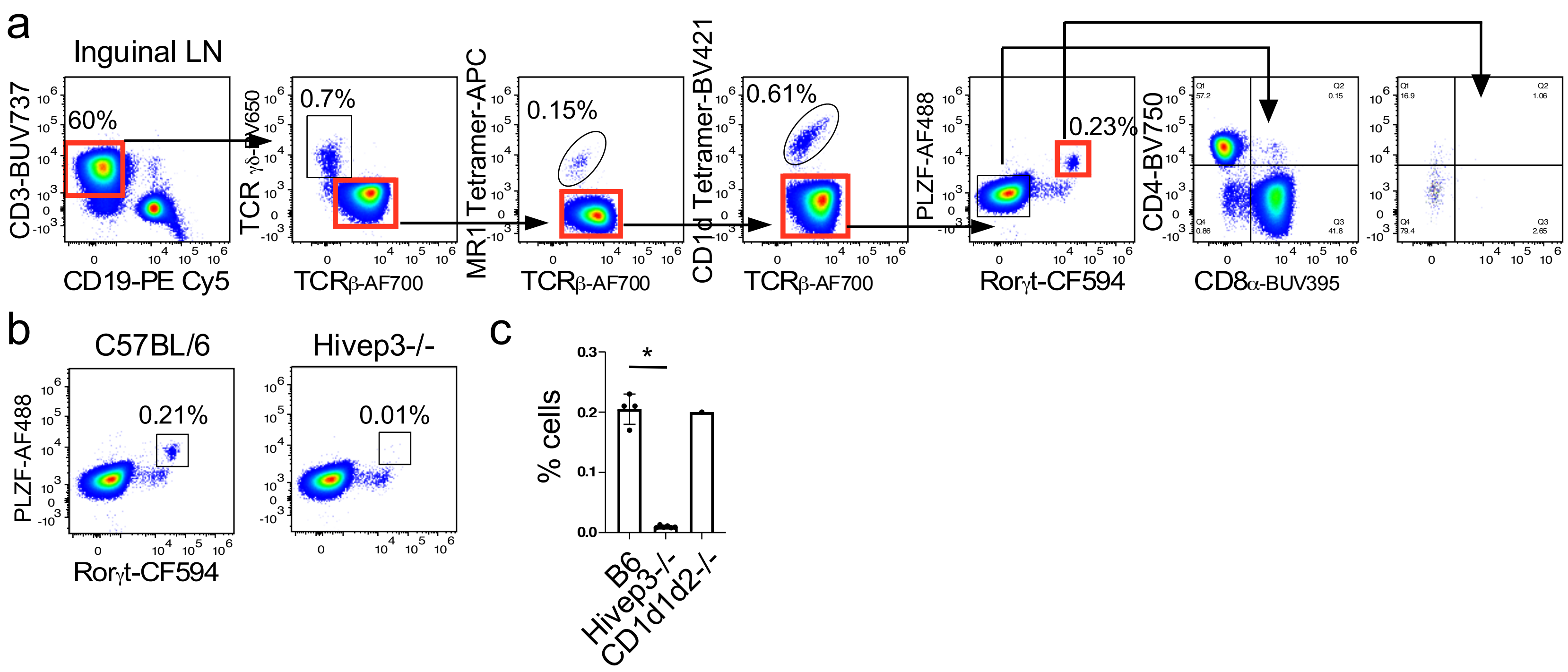

Supplementary Figure 9. Identification of $CD4^-CD8^-$ $TCR\beta^+$ T cell population that co-express PLZF and Rorγt in the inguinal lymph nodes of mice. (**a**) Gating strategy used to identify non-iNKT, non-MAIT, αβ T cells with co-expression of PLZF and Rorγt. (**b**) Representative analysis of PLZF and Rorγt expression in non-iNKT, non-MAIT αβ T cells in the inguinal lymph nodes of C57BL/6 and *Hivep3*$^{-/-}$ mice. (**c**) Quantified proportions of $PLZF^+$ $Ror\gamma t^+$ non-iNKT, non-MAIT αβ T cells in the inguinal lymph nodes of C57BL/6, *Hivep3*$^{-/-}$ and CD1d1d2$^{-/-}$ mice. Data are from two independent experiments with a total of 4 C57BL/6, 5 Hivep3$^{-/-}$ and 1 CD1d1d2$^{-/-}$ mice with 8-10 weeks old mice of both sexes used. p=0.0159 between C57BL/6 and Hivep3$^{-/-}$ using the two-tailed Mann–Whitney U test. Data are mean ± SD with dots representing individual values. Source data are provided as a source data file.

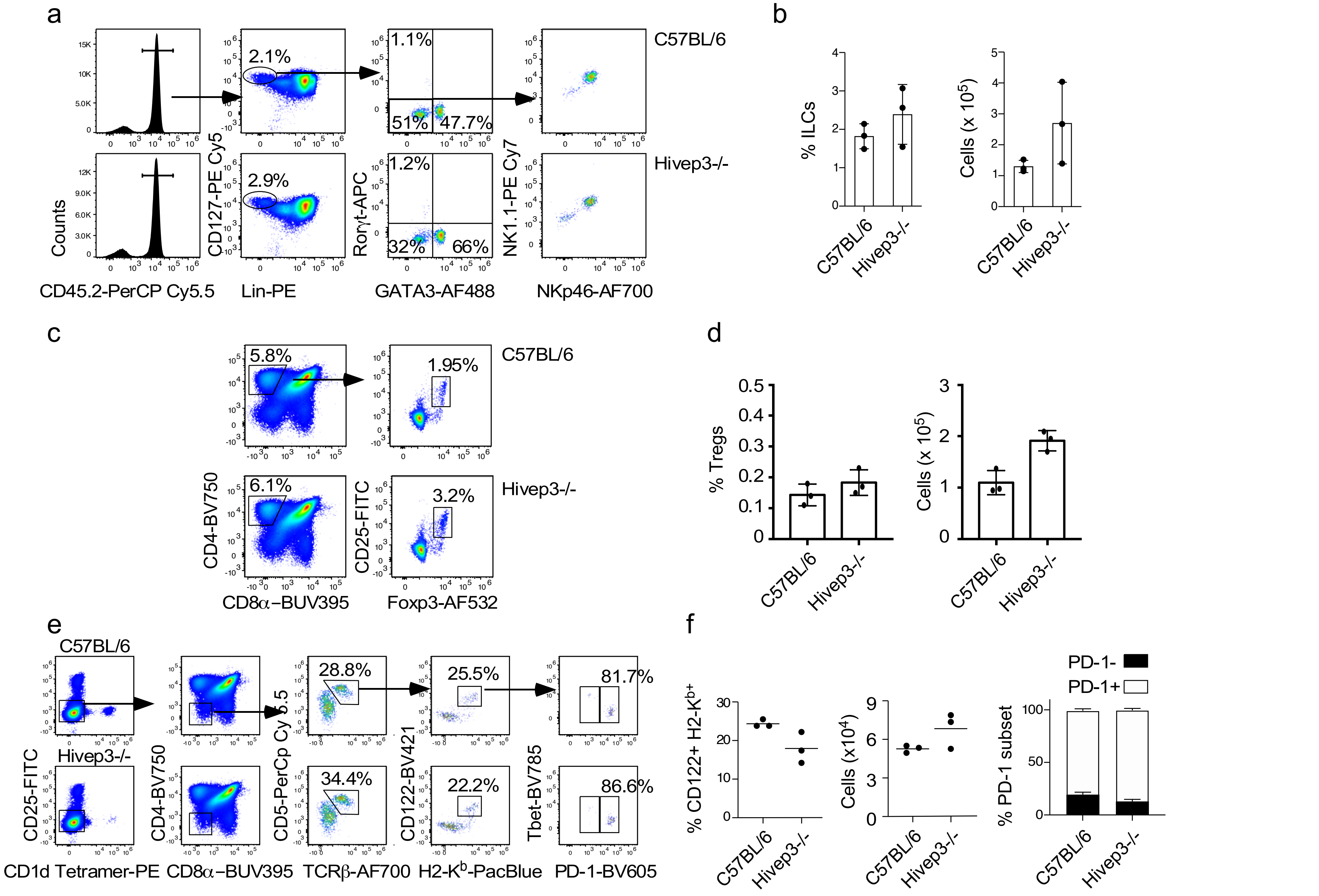

Supplementary Figure 10. Analysis of innate lymphoid cells (ILCs), regulatory T cells and intraepithelial lymphocyte precursors in C57BL/6 and *Hivep3*$^{-/-}$ mice. (**a**) Gating strategy used to identify and quantify the proportions of ILC populations in the lungs of C57BL/6 and *Hivep3*$^{-/-}$ mice. (**b**) Quantified proportions and total numbers of ILCs in the lungs of C57BL/6 and *Hivep3*$^{-/-}$ mice. (**c**) Gating strategy used to identify and quantify the proportions Tregs in the thymus of C57BL/6 and *Hivep3*$^{-/-}$ mice. (**d**) Quantified proportions and total numbers of Tregs in the thymus of C57BL/6 and *Hivep3*$^{-/-}$ mice. (**e**) Flow cytometry for the identification of IELp cells in the thymus. Numbers in plots indicate percent cells in outlined area. (**f**) summary of the frequency and number of mature CD122^{+} H-2K^{b+} cells gated as in (**e**) and total PD-1^{+} cells and PD-1^{-} in the thymic IELp population of C57BL/6 and *Hivep3*$^{-/-}$ mice. Data are from one experiment with a total of 3 mice per group. Data in the figure are mean ± SD with dots representing individual values. Source data are provided as a source data file.

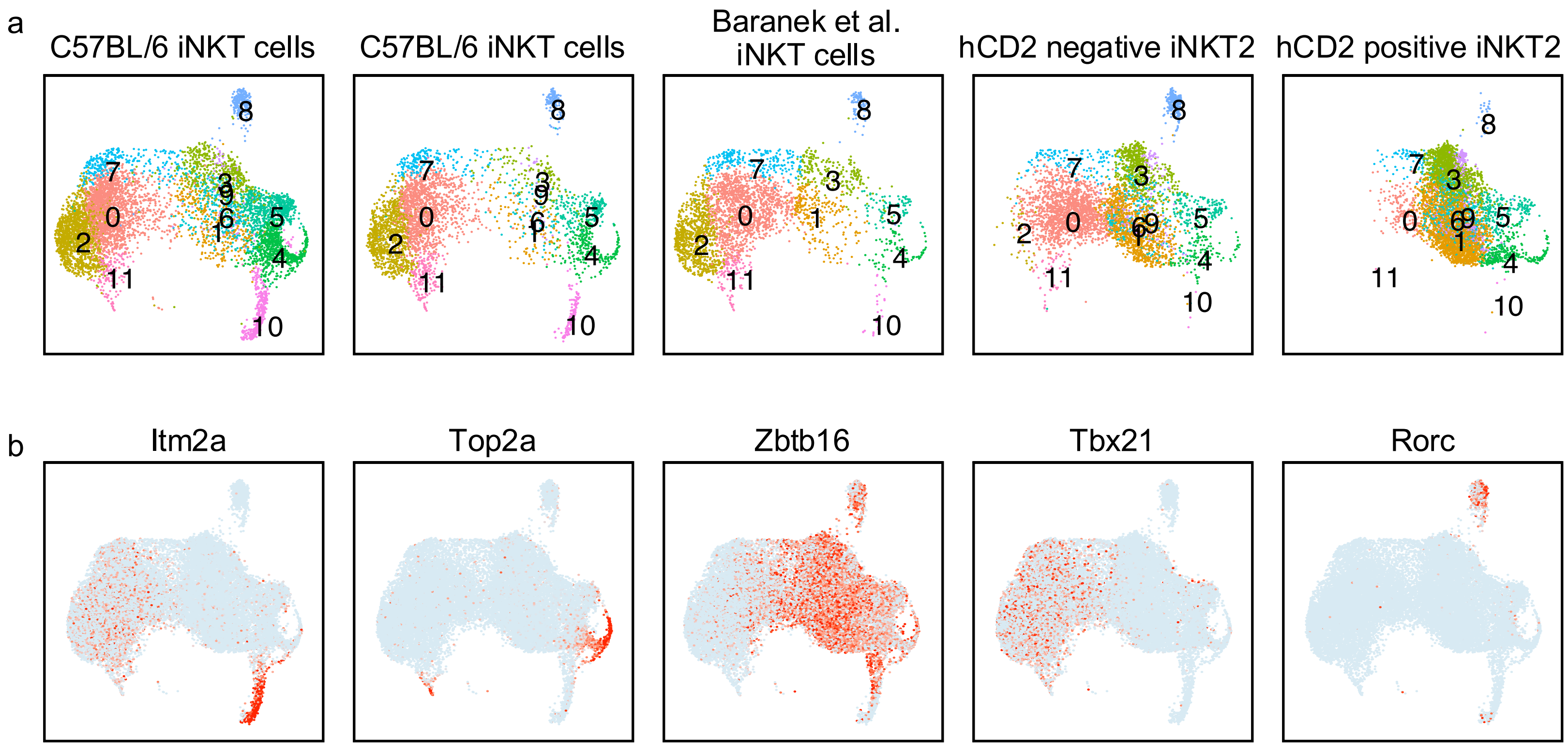

Supplementary Figure 11. scRNA-seq analysis of steady-state C57BL/6 thymic iNKT cells. (**a**) Uniform manifold approximation and projection (UMAP) of two independent scRNA-seq data sets from C57BL/6 thymic iNKT cells, C57BL/6 thymic iNKT cells from Baranek et al. (Ref 52) and from hCD2neg and hCD2pos iNKT2 cells (CD122^{-} ICOS^{+} CD138^{-}) sorted from the thymus of BALB/c KN2 mice and integrated using FastMNN. (**b** Expression of five typical genes used to define most common iNKT cell subsets and cycling cells.

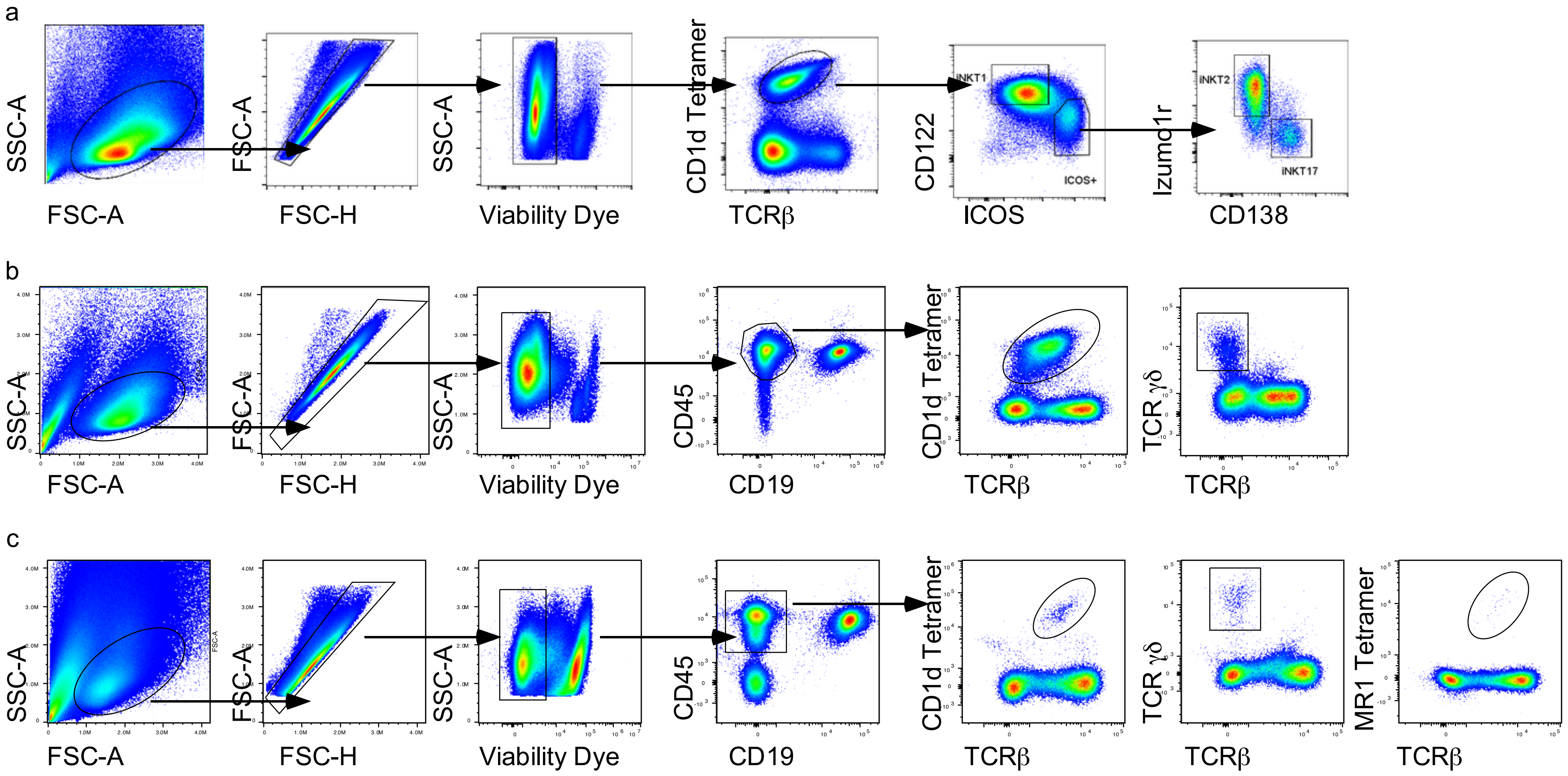

Supplementary Figure 12. Gating strategies implemented to identify T_{inn} populations for analyses and sorting. (a) Representative Gating strategy used for sorting thymic iNKT cell subsets submited to ATAC-seq. (b) Representative Gating strategy in the liver (c) Representative Gating strategy in the lungs
