# Supplementary material for: Thymic iNKT single cell analyses unmask the common developmental program of mouse innate T cells
Source: Nat Commun. 2020 Dec 7;11:6238. doi: 10.1038/s41467-020-20073-8 (PMC7721697; doi:10.1038/s41467-020-20073-8)
Supplement: Supplementary file 3 — Description of Additional Supplementary Files [file 41467_2020_20073_MOESM3_ESM.pdf]

Description of Additional Supplementary Files

File Name: Supplementary Data 1
Description: A list of genes that are differentially expressed in each of the 11 thymic C57BL/6 iNKT clusters. Only differentially expressed genes enriched in each cluster of cells with an adjusted p-value (using the Benjamini-Hochberg correction) below 0.05 were retained.

File Name: Supplementary Data 2
Description: A list of genes that are differentially expressed between C57BL/6 and Hivep3-/- iNKT cells for each of the 10 clusters. Only differentially expressed genes between strains within each cluster with an adjusted p-value (using the Benjamini-Hochberg correction) below 0.05 were retained.

File Name: Supplementary Data 3
Description: A list of genes that are differentially expressed in each of the 12 clusters yet conserved between C57BL/6 thymic iNKT and MAIT cells. Only differentially expressed genes enriched in each cluster of cells with an adjusted p-value (using the Benjamini-Hochberg correction) below 0.05 were retained.
