# Supplementary material for: Thymic iNKT single cell analyses unmask the common developmental program of mouse innate T cells
Source: Nat Commun. 2020 Dec 7;11:6238. doi: 10.1038/s41467-020-20073-8 (PMC7721697; doi:10.1038/s41467-020-20073-8)
Supplement: Supplementary file 7 — Reporting Summary [file 41467_2020_20073_MOESM7_ESM.pdf]

nature research

Corresponding author(s): Laurent Gapin

Last updated by author(s): 11/01/2020

Reporting Summary

Nature Research wishes to improve the reproducibility of the work that we publish. This form provides structure for consistency and transparency in reporting. For further information on Nature Research policies, see our Editorial Policies and the Editorial Policy Checklist.

Statistics

For all statistical analyses, confirm that the following items are present in the figure legend, table legend, main text, or Methods section.

n/a	Confirmed	
☐	☒	The exact sample size (*n*) for each experimental group/condition, given as a discrete number and unit of measurement
☐	☒	A statement on whether measurements were taken from distinct samples or whether the same sample was measured repeatedly
☐	☒	The statistical test(s) used AND whether they are one- or two-sided<br>*Only common tests should be described solely by name; describe more complex techniques in the Methods section.*
☐	☒	A description of all covariates tested
☐	☒	A description of any assumptions or corrections, such as tests of normality and adjustment for multiple comparisons
☐	☒	A full description of the statistical parameters including central tendency (e.g. means) or other basic estimates (e.g. regression coefficient) AND variation (e.g. standard deviation) or associated estimates of uncertainty (e.g. confidence intervals)
☐	☒	For null hypothesis testing, the test statistic (e.g. *F*, *t*, *r*) with confidence intervals, effect sizes, degrees of freedom and *P* value noted<br>*Give P values as exact values whenever suitable.*
☒	☐	For Bayesian analysis, information on the choice of priors and Markov chain Monte Carlo settings
☒	☐	For hierarchical and complex designs, identification of the appropriate level for tests and full reporting of outcomes
☒	☐	Estimates of effect sizes (e.g. Cohen's *d*, Pearson's *r*), indicating how they were calculated

Our web collection on statistics for biologists contains articles on many of the points above.

Software and code

Policy information about availability of computer code

Data collection	BD FACSDiva Software (v8.0) for flow cytometry
Data analysis	scRNA-seq: FastQC v0.11.9, cellRanger v3.1, custom scripts in R (v3.6.1; packages: Seurat v3.1.4, Slingshot v1.4.0, clusterProfiler v3.0.4, monocle3 0.2.0); Flow cytometry: FlowJo v10; ATAC-seq: bcl2fastq (v2.20), Trimmomatic v0.36, custom scripts in R (v3.6.1; packages: chromVAR v1.10.0), HOMER (v4.11 on a Mac); Other figures: Graphpad Prism v8; Custom code: All the custom code used for processing and analyzing the data in the study were compiled into a single publicly available GitHub repository [https://github.com/krovi137/NKT-Single-Cell-Analysis/tree/main].

For manuscripts utilizing custom algorithms or software that are central to the research but not yet described in published literature, software must be made available to editors and reviewers. We strongly encourage code deposition in a community repository (e.g. GitHub). See the Nature Research guidelines for submitting code & software for further information.

Data

Policy information about availability of data

All manuscripts must include a data availability statement. This statement should provide the following information, where applicable:

- Accession codes, unique identifiers, or web links for publicly available datasets
- A list of figures that have associated raw data
- A description of any restrictions on data availability

Data that support the findings of this study have been deposited in NCBI GEO with the accession code GSE152786 and GSE160518. All the other data are included within the article, or supplemental information or available from the authors upon reasonable requests.

Field-specific reporting

Please select the one below that is the best fit for your research. If you are not sure, read the appropriate sections before making your selection.

☒ Life sciences ☐ Behavioural & social sciences ☐ Ecological, evolutionary & environmental sciences

For a reference copy of the document with all sections, see nature.com/documents/nr-reporting-summary-flat.pdf

Life sciences study design

All studies must disclose on these points even when the disclosure is negative.

Sample size	Sequencing experiments were performed in duplicate. As shown in Figures 5b and 7a, there is a high degree of reproducibility between our biological duplicate samples for both scRNA-seq and ATAC-seq data.<br><br>Sample sizes for mouse experiments were not predetermined. In each mouse experiment, we analyzed a minimum of 3 biological replicates from at least 2 independent experiments. The high reproducibility of the data was sufficient to justify these numbers. Additionally, these numbers of biological replicates were sufficient to obtain significant differences between different samples in a statistically rigorous manner.
Data exclusions	For scRNA-seq computational analyses, low quality cells were filtered to ensure that at least 500 different transcripts were found. Additionally, to exclude potential doublets, we removed cells that contained more than 4100 transcripts, as this was significantly greater than the number of transcripts found in a high proportion of cells. Lastly, to ensure that the cells we analyzed displayed high viability, we excluded cells that contained > 6% mitochondrial transcripts (high levels of mitochondrial transcripts in cells are indicative of cells undergoing cell death).<br><br>For flow cytometry analyses, doublet cells and cells that stained with a viability dye were excluded from downstream analyses. These exclusion criteria were pre-established.<br><br>For ATAC-seq analyses, regions of the genome that are blacklisted by ENCODE were excluded from the analyses. Additionally, regions mapping to the Y chromosome were also excluded to remove any sex-specific biases. These exclusion criteria were pre-established.
Replication	For the scRNA-seq data, we generated 2 thymic iNKT datasets for each mouse strain. The data showed minor differences, suggesting that our findings are highly reproducible. Similarly, for the ATAC-seq datasets, we generated distinct biological duplicate samples for each thymic iNKT subset from both WT and KO thymi that are highly reproducible.<br><br>For flow cytometry experiments, the data were reproduced in multiple individual mice, usually from 2-3 independent experiments. The repeated experiments corroborated the initial findings, once again indicating that the data generated are highly replicable. The number of times an experiment was conducted is included in the figure legend corresponding to each figure.
Randomization	Samples belonging to a given experimental group/genotype were randomly allocated. For the various ex vivo experiments, mice were age-matched to minimize age-dependent differences. No other randomizations were performed as this was not a case-control study
Blinding	Investigator were not blinded to experimental groups or genotype for experiments after processing as this would preclude grouping of biological replicates for analysis.

Reporting for specific materials, systems and methods

We require information from authors about some types of materials, experimental systems and methods used in many studies. Here, indicate whether each material, system or method listed is relevant to your study. If you are not sure if a list item applies to your research, read the appropriate section before selecting a response.

Materials & experimental systems

n/a	Involved in the study	
☐	☒	Antibodies
☒	☐	Eukaryotic cell lines
☒	☐	Palaeontology and archaeology
☐	☒	Animals and other organisms
☒	☐	Human research participants
☒	☐	Clinical data
☒	☐	Dual use research of concern

Methods

n/a	Involved in the study	
☒	☐	ChIP-seq
☐	☒	Flow cytometry
☒	☐	MRI-based neuroimaging

Antibodies

Antibodies used	Surface antibodies:

from BioLegend – CD3 (clone 17A2; Catalog #100204, Catalog #100218), CD4 (clone GK1.5; Catalog #100447), CD4 (clone RM4-5; Catalog #100557), CD5 (clone 53-7.3; Catalog #100624), CD19 (clone 6D5; Catalog #115509, Catalog #115521, Catalog #115528), CD25 (clone PC61; Catalog #102006), CD44 (clone IM7; Catalog #103044), CD45.1 (clone A20; Catalog #110714), CD45.2 (clone 104; Catalog #109827, Catalog #109828), CD69 (clone H1.2F3; Catalog #104530), CD81 (clone Eat-2; Catalog #104913), CD127 (clone SB/199; Catalog #121123), CD138 (clone 281-2; Catalog #142531), H-2Kb (clone AF6-88.5; Catalog #116513), ICOS (clone C398.4A; Catalog #313530), ICOS (clone 7E.17G9; Catalog #117406, Catalog #117424), Izumo1r (also called FR-4; clone 12A5; Catalog #125012), Lineage cocktail (clones 145-2C11, RB6-8C5, RA3-6B2, Ter-119 and M1/70; Catalog #133303), Ly-6C (clone HK1.4; Catalog #128029, Catalog #128030), NK1.1 (clone PK136; Catalog #108713, Catalog #108730, Catalog #108736, Catalog #108753), PD-1 (clone 29F.1A12; Catalog #135206, Catalog #135220), TCR (clone H57-597; Catalog #109220, Catalog #109224, Catalog #109230) and V1 (clone 2.11; Catalog #141104, Catalog #141108, Catalog #141112); from BD Biosciences – CCR9 (clone CW-1.2; Catalog #565412), CD3 (clone 145-2C11; Catalog #612771), CD4 (clone H129.19; Catalog #747275), CD8 (clone 53-6.7; Catalog #563786), CD24 (clone M1/69; Catalog #563545, Catalog #612953), CD122 (clone TM-1; Catalog #562960), NKp46 (clone 29A1.4; Catalog #561169), TCR (clone GL3; Catalog #563993) and V6.3 (clone 8F4H7B7; Catalog #555321); from Thermo Fisher Scientific – CD122 (clone TM-1; Catalog #50-245-876).

Intracellular antibodies:
from Abcam – Lef-1 (clone EPR2029Y; Catalog #ab137872); from BioLegend – Bcl-2 (clone BCL/10C4; Catalog #633512), BrdU (clone 3D4; Catalog #364110, Catalog #364114), GATA3 (clone 16E10A23; Catalog #653808), Helios (clone 22F6; Catalog #137222), IFN (clone XMG1.2; Catalog #505810, Catalog #505826, Catalog #505830), IL-4 (clone 11B11; Catalog #504104), PLZF (clone 9E12; Catalog #145808), Granzyme B (clone GB11; Catalog #515408) and T-bet (clone 4B10; Catalog #644816, Catalog #644824, Catalog #644835); from BD Biosciences – RORt (clone Q31-378; Catalog #562684); from Miltenyi Biotec – Tox (clone REA473; Catalog #130-118-335); from Thermo Fisher Scientific – Egr2 (clone erongr2; Catalog #12-6691-82, Catalog #17-6691-82, Catalog #25-6691-82), FoxP3 (clone FJK-16s; Catalog #58-5773-82), Granzyme A (clone GzA-3G8.5; Catalog #17-5831-82), Granzyme B (clone NGZB; Catalog #48-8898-82), Ki-67 (clone SolA15; Catalog #25-5698-82, Catalog #48-5698-82), Granzyme A (clone GzA-3G8.5, Catalog #17-5831-82), PLZF (clone Mags.21F7; Catalog #53-9320-82, Catalog #25-9322-82) and RORt (clone B2D; Catalog #17-6981-82).

Validation

Antibodies are commercially available and were validated and titrated by using positive and negative control cells before using them in any of the reported experiments. The manufacturer's validations on their websites were also used as guidelines. For cytokine staining, FMO (fluorescence minus one) and isotype controls were also used to ensure a positive signal.

Animals and other organisms

Policy information about studies involving animals; ARRIVE guidelines recommended for reporting animal research

Laboratory animals

C57BL/6 mice: males and females were both used between 8-10 weeks of age.
Hivep3 KO mice backcrossed to a C57BL/6 background - males and females were both used between 8-10 weeks of age.
CD45.1 congenic mice crossed to C57BL/6 background - males and females were used between 8-10 weeks of age.

Experimental mice were euthanized using the gradual fill method with CO2 prior to tissue harvest and downstream analyses. All mice were raised in a specific pathogen-free environment, ambient temperature 23 +/- 1 degC, humidity 50 +/- 10% and a light/dark cycle of 12 hours at the Office of Laboratory Animal Research at the University of Colorado Anschutz Medical campus.

Wild animals

The study did not involve wild animals.

Field-collected samples

The study did not involve samples collected from the field.

Ethics oversight

All animal procedures were approved by the UCD Institutional Animal Care and Use Committees and were carried out in accordance with the approved guidelines.

Note that full information on the approval of the study protocol must also be provided in the manuscript.

Flow Cytometry

Plots

Confirm that:

☒ The axis labels state the marker and fluorochrome used (e.g. CD4-FITC).

☒ The axis scales are clearly visible. Include numbers along axes only for bottom left plot of group (a 'group' is an analysis of identical markers).

☒ All plots are contour plots with outliers or pseudocolor plots.

☒ A numerical value for number of cells or percentage (with statistics) is provided.

Methodology

Sample preparation

For thymic iNKT isolations, thymocytes were enriched for PBS57-CD1d reactive cells by incubating thymocyte cell suspensions with PE or APC conjugated PBS57-CD1d tetramers for 45 minutes at 4°C, then incubated with anti-PE or anti-APC magnetic microbeads (Miltenyi Biotec) for 15 minutes at 4°C, followed by separation by using an autoMACS Pro Separator (Miltenyi Biotec) according to manufacturer's instructions. Subsequently, cells in the positive fraction were first stained for surface markers followed by staining for intracellular markers before being subjected to flow cytometric acquisition.

To prepare lung single-cell suspensions, lungs were finely chopped with scissors in a 48 well plate and treated with 3 µg ml-1 collagenase III (Worthington, Lakewood, NJ), 5 µg ml-1 DNAse, and complete media (RPMI containing 10% fetal calf serum and enriched supplements) for 60 min at 37°C with gentle pipetting at 20 min intervals. Cells were then filtered through a 70 µm cell strainer and washed with complete media.

For liver single cell preparations, leukocytes were isolated by cutting individual livers into small pieces and gently pressed through a 70 µm filter placed on top of a 50 ml falcon tube and resuspended in FACS buffer (PBS, 0.5% BSA, 0.5 mM EDTA, 1% Azide). The cells were washed twice in ice-cold FACS buffer and spun through 33.8% Percoll (Amersham Pharmacia Biotech) for 12 min at 2000 rpm, room temperature. Red blood cells were removed by resuspending the pellet in red blood cell lysis buffer for 5 min at room temperature and washed with complete media. Cells were resuspended in FACS buffer and filtered through a 40 µm cell strainer. Approximately 2 x 106 cells were filtered (40 µm) and used for flow cytometric analysis.

Instrument	The stained cells were then analyzed on a BD LSRFortessa (BD Biosciences) or Cytek Aurora (Cytek).
Software	Flow cytometric data was collected using the BD FACSDiva (BD Biosciences) software. Data were processed with FlowJo software v10 (TreeStar).
Cell population abundance	Sorted cells displayed high (>99%) purity. This was determined in two different ways. Firstly, a small sample of the sorted cells was analyzed using a cytometer to confirm that the sorted population was pure. Secondly, since scRNA-seq was performed following some of our sorts, we could identify with single cell resolution the extent of purity within our post-sort population. We could readily confirm by transcript expression that our sorted cells were of high purity in this manner.
Gating strategy	For bulk iNKT cell analysis, cells were initially gated on FSC-A/SSC-A, followed by doublet exclusion using FSC-H/FSC-A. To ensure we analyzed only live cells, we would then gate on cells that stained negative for a viability dye. Lastly, bulk iNKT cells were determined by gating on cells that stained for both PBS57-CD1d tetramer and the TCRb antibody.<br><br>For iNKT subset analysis, iNKT cells were initially identified as described. Then, for transcription factor analysis, the iNKT cell population was subdivided by expression of the transcription factors PLZF and RORgt (as depicted in Figure 2e). This allowed us to identify the 3 thymic iNKT subsets. For identification of iNKT subsets by surface markers, iNKT subsets were distinguished by their differential expression of CD122, ICOS, Izumo1r and CD138 - iNKT1 cells are ICOSlow CD122+, iNKT2 cells are ICOS+ CD138- Izumo1r+ and iNKT17 cells are ICOS+ CD138+ Izumo1r-. This is depicted in Supplementary Figure 2.<br><br>For many other analyses involving other markers/cytokines, positive and negative staining was established using either isotype control antibodies or unstimulated control samples.

☒ Tick this box to confirm that a figure exemplifying the gating strategy is provided in the Supplementary Information.
